# Sarcomatoid Renal Cell Carcinoma in an Adolescent with Sickle Cell Anaemia

**DOI:** 10.1155/2017/2129450

**Published:** 2017-10-31

**Authors:** H. R. Ahmad, J. A. Faruk, M. A. Bugaje, A. Solomon, M. O. A. Samaila, R. M. Akuse

**Affiliations:** ^1^Haematology/Oncology Unit, Department of Paediatrics, Ahmadu Bello University/Teaching Hospital, Zaria, Nigeria; ^2^Nephrology Unit, Department of Paediatrics, Ahmadu Bello University/Teaching Hospital, Zaria, Nigeria; ^3^Department of Pathology, Ahmadu Bello University/Teaching Hospital, Zaria, Nigeria

## Abstract

Malignancies have been reported to occur in people with sickle cell disease. Renal medullary carcinoma (RMC), also tagged seventh sickle cell nephropathy, is an aggressive cancer seen almost exclusively in people with sickle cell disease with more than 160 cases reported worldwide, but only few cases were reported in patients with sickle cell anaemia (HBSS) and from Nigeria. Sarcomatoid renal cell carcinoma is a renal tumour of any histologic variant containing foci of high-grade malignant spindle cells. We report an adolescent girl with sickle cell anaemia (HBSS) who presented with left renal tumour, histology of which confirmed a diagnosis of sarcomatoid renal cell carcinoma (sRCC). Surgical debulking and palliative care with chemotherapy were given, and she demised 10 months after. The rarity of the case and challenges of managing a cancer in the background of a chronic haematologic disorder are highlighted.

## 1. Introduction

Malignancies do occur in individuals with sickle cell disease (SCD), mostly in haemoglobin phenotypes HbSS, HbSC, and HbS/*β*-thal with an incidence of 1.74/1000 patient years [1, 2], and the risk factors are similar to that in the general population. Chronic renal medullary hypoxia from low oxygen tension, high acidity, and high osmolality encourage chronic stasis of sickled cells leading to renal capillary occlusion, tubular epithelial hypertrophy, and hyperproliferation that eventually results in tumourigenesis through the activation of hypoxia-inducible factor 1 pathway [3, 4]. Behrman attributed six types of nephropathies in SCD to chronic renal medullary hypoxia: gross haematuria, papillary necrosis, nephrotic syndrome, renal infarction, hyposthenuria, and pyelonephritis, while more recently, RMC was described as the seventh sickle cell nephropathy [5].

Renal medullary carcinoma (RMC) is a highly aggressive tumour found almost exclusively in people with SCD, classically male, black young adults with SCT [5, 6]. Patients present with haematuria, loin pain, weight loss, and abdominal swelling. Although there are now reports of patients that responded favourably to both chemotherapy [7, 8] and radiation for central nervous system relapse [9], majority of symptomatic patients already have retroperitoneal lymphadenopathy and organ metastasis by the time of diagnosis with fatal outcomes [4, 5, 7, 10, 11]. The estimated life expectancy from the time of diagnosis is about 15 months. The relative young age at presentation and the exclusiveness of the disease to people with SCD suggest a genetic origin closely linked to that for SCD [10]. sRCC on the other hand is a renal tumour of any histologic variant containing foci of high-grade malignant spindle cells and has not been shown to have a predilection for SCT [11].

Although Nigeria has the highest global burden of SCD, a multicenter national survey for renal tumours in SCD documented a 10-year prevalence of 0.056% for RMC [12].

We present an adolescent girl with sickle cell anaemia who developed sRCC distinct from RMC as the first case report of such a tumour in Zaria, Nigeria, to showcase the rarity of the tumour and management challenges encountered.

## 2. Case Presentation

MR was a 17-year-old girl diagnosed with HbSS at the age of 2 years following jaundice, fever, and bone pain. She had dactylitis at 2 1/2 years and was on regular follow-up with an average of one pain crisis per year until 10 years of life when she had two admissions, managed as typhoid fever and osteomyelitis, respectively. She subsequently had 10 vaso-occlusive episodes over the next 6 years, managed as outpatient and never had blood transfusion up to that point. She had normal growth and attained menarche at 15 years. Her routine medications included folic acid, penicillin, and proguanil.

She presented with fever, left loin pain, dysuria, and urine frequency at age 16 years with associated left renal angle tenderness, during which urinalysis showed significant proteinuria and positive leucocyte esterase, pyuria, but no organism was isolated presumably because of prior antibiotic use. She was treated for pyelonephritis with full resolution of symptoms. Further investigations including abdominopelvic ultrasonography and intravenous urography (IVU) revealed a nonexcreting left kidney. She defaulted from follow-up and presented 16 months later with haematuria and left loin swelling in addition to the left loin pain which had persisted.

A CT urogram revealed a huge, lobulated, heterogeneous, complex left renal mass 16 cm × 11 cm on coronal section and 12.4 cm × 9.6 cm on axial view with cystic components, septations, and no excretion of contrast from the same kidney. A diagnosis of left renal tumour was made, and she had left cytoreductive nephrectomy and lymph node excision. Intraoperatively, the tumour measured 25 cm × 16 cm × 12 cm, infiltrating the posterior abdominal wall muscles and the left renal vein. There were also five huge para-aortic lymph nodes, measuring 2–5 cm with metastatic masses involving the liver, lung, and spleen. The tumour was staged at T4N2M1. She had an uneventful postsurgical recovery.

**Figure 1:**
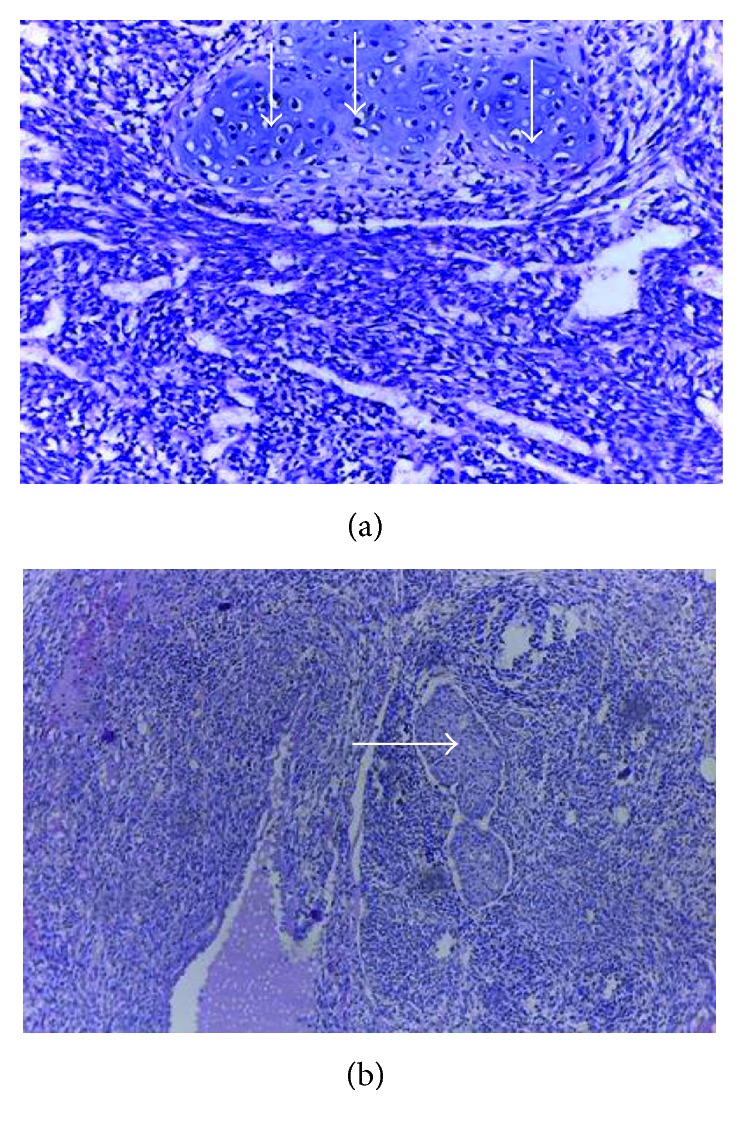
(a) Biphasic tumour, composed of epithelial components dispersed in tubules and microcysts whose lining was flattened to cuboidal epithelium having vesicular nuclei and scanty cytoplasm. (b) The mesenchymal component was dispersed in sheets, fascicles, and focal storiform whose comprised cells were spindle-shaped with vesicular nuclei and scanty to moderate cytoplasm. H&E staining, magnification, ×40.

Histological examination findings leading to confirmation of sRCC of the left kidney are shown in Figure 1.

**Figure 2:**
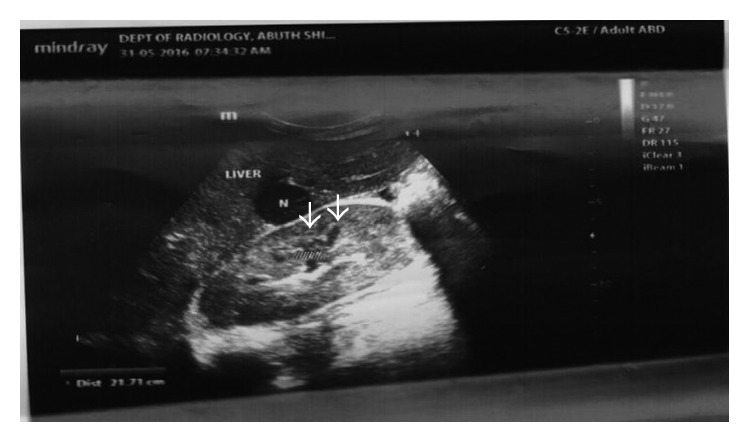
Abdominal ultrasound showing liver metastasis.

**Figure 3:**
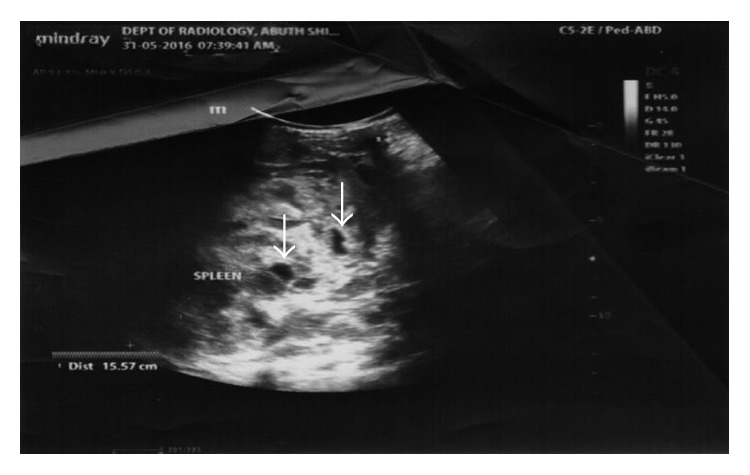
Abdominal ultrasound showing splenic metastasis.

She had palliative care comprising analgesics including morphine, antibiotics, blood transfusions, granulocyte colony-stimulating factors, allopurinol, cytotoxic chemotherapy (doxorubicin 20 mg/m^2^ and vincristine 1.5 mg/m^2^ at 2 weekly intervals initially and 4 weekly when renal function deteriorated), and hydroxyurea 10 mg/kg daily for the sickle cell. Over the next 10 months, her management was progressively challenged by recurrent anaemia (packed cell volume ranges between 9% and 24%), worsening of renal function evidenced by rising urea from 5.6 to 34 mmol/l, rising creatinine from 59 umol/l to 312 umol/l, reducing glomerular filtration rate (GFR) from 98 to 19 ml/min/1.73 m^2^, increasing uric acid from 141 to 560 umol/l, and further dissemination of the tumour on the left renal bed with more hepatic (Figure 2) and splenic (Figure 3) metastatic deposits which led to her demise.

## 3. Discussion

A case of sRCC in a girl with SCA is hereby presented. Although she remained stable up to 10 years of age, crisis frequency increased thereafter including 2 admissions within a year. Left pyelonephritis and subsequent evaluations would have led to an early definitive diagnosis had she not defaulted from follow-up. Overall, diagnosis was made 2 years later when metastasis to the liver, spleen, and lungs had already occurred.

sRCC has poor response to both chemotherapy even though some workers documented complete response to doxorubicin-based regimens [13]. Advanced stage at diagnosis, presence of a poorly functioning single kidney, and SCA status made the decision to attempt treatment even more difficult. Palliation with drugs with minimal effect on renal function was used due to lack of more efficacious treatments and protocol. Treatment of SCA with hydroxyurea temporarily reduced the need for blood transfusion in addition to erythropoietin and folinic acid. The disease progression and worsening renal function necessitated continuation of only supportive care in the form of blood transfusions, analgesia, nutrition, and multivitamins.

As sRCC occurs in any histologic renal cancer subtype, perhaps the background histology for this patient was RMC but could be any histologic type.

Although most patients with RMC were male, black young adults, with SCT, and had right side affectation [14], our patient was an adolescent girl with HBSS and left kidney affectation. The presentation with loin pain, haematuria, and abdominal swelling is consistent with other reported cases. All the six cases in the review by Avery et al. and Davies series had metastasis at diagnosis, with an average time to diagnosis of 4.7 months and time from onset of symptoms to death of 7.7 months [5, 14]. The time to diagnosis for our patient was 2 years, and she lived for 10 months thereafter. Five of six patients reported by Avery et al. had nephrectomy, and all had some chemotherapy with poor response and early demise [14]. The most common sites of metastasis are the lungs, lymph nodes, liver, and bone as in our patient [8–10, 14, 15].

The young age at presentation for this tumour suggests underlying genetic abnormality(ies), and the occurrence of the disease almost exclusively in people with haemoglobinopathies might suggest a genetic anomaly involving chromosome 11 since the gene for beta globin is at the terminal portion of its short arm [14, 15].

Despite the high burden of SCD in Nigeria with prevalence rate of SCT at 25%, there is no report of RMC or sRCC in the literature of this tumour apart from the 2 cases mentioned from Kano in the survey by Anazaoze et al. [12].

## 4. Conclusions

Although the incidence of renal neoplasms is generally very low, the occurrence in subjects with haemoglobinopathies presents peculiar challenges in management and may be associated with higher morbidity and mortality. The need for close monitoring and early evaluation of SCD patients especially if presenting with one or more features of nephropathy is strongly recommended.
